# The function of oxytocin: a potential biomarker for prostate cancer diagnosis and promoter of prostate cancer

**DOI:** 10.18632/oncotarget.16107

**Published:** 2017-03-10

**Authors:** Huan Xu, Shi Fu, Qi Chen, Meng Gu, Juan Zhou, Chong Liu, Yanbo Chen, Zhong Wang

**Affiliations:** ^1^ Department of Urology, Shanghai 9th People's Hospital, Shanghai Jiaotong University School of Medicine, Shanghai 200011, China

**Keywords:** prostate cancer, oxytocin, APPL1, metabolism, proliferation

## Abstract

**Purpose:**

To measure the level of oxytocin in serum and prostate cancer (PCa) tissue and study its effect on the proliferation of PCa cells.

**Results:**

Oxytocin level in serum was significantly increased in PCa patients compared with the no-carcinoma individuals. Additionally, the levels of oxytocin and its receptor were also elevated in the PCa tissue. However, no significant difference existed among the PCa of various Gleason grades. Western blot analysis confirmed the previous results and revealed an increased expression level of APPL1.

**Materials and methods:**

The level of oxytocin in serum was measured by ELISA analysis. The expression of oxytocin and its receptor in prostate was analyzed by immunohistochemistry. The proliferation and apoptosis of PCa cells were assessed by the Cell Counting Kit 8 (CCK8) assay, cell cycle analysis and caspase3 activity analysis, respectively. Western blot analysis was used for the detection of PCNA, Caspase3 and APPL1 protein levels.

**Conclusions:**

Serum and prostatic oxytocin levels are increased in the PCa subjects. Serum oxytocin level may be a biomarker for PCa in the future. Oxytocin increases PCa growth and APPL1 expression.

## INTRODUCTION

Prostate cancer (PCa) is one of the most common cancers in males in Europe and North America, whose morbidity has been steadily increasing in recent years in Asian countries [1]. In addition, it is the most common cause of cancer death in men after lung and colon carcinoma [2, 3]. Among the therapies available to treat PCa, androgen deprivation is one of the standard treatments for advanced PCa. However, almost all advanced PCa become resistant to androgen deprivation and inevitably turn into castration resistant prostate cancers (CRPC) which eventually lead to death [4]. Despite the high death rate, only a small number of drugs have been brought to the clinic. Metabolic disorders are widely recognized as important contributors to the pathological process of PCa. Among them, metabolic syndrome (MetS), obesity, hyperlipidemia and diabetes are influencing factors on the growth and development of PCa. It is known that the insulin resistance (IR) is one of the main factor linking MetS and PCa [5, 6]. Moreover, some hormones changed in the MetS patient, such as adiponectin, leptin and GLP-1, regulate epithelial cells proliferation, cancer cells migration, angiogenesis and apoptosis inhibition [7–9]. Accordingly, MetS cannot be ignored as a new research area for PCa treatment. The central endocrine system involved in PCa progression comprises the hypothalamus, the pituitary gland and the prostate gland. The hypothalamic–pituitary–gonadal (HPG) axis regulates the key feedback loops involved in the biosynthesis of testosterone (T) which plays an important role in the PCa progression [10, 11].

Oxytocin (OT) is a hypothalamic hormone. Among its various widely accepted functions, the main is in uterine contractions and milk secretion. However, current studies focus more and more on its effect on the metabolism regulation and tumor promotion effects. Remarkably, OT and its receptor were found to be present in both epithelial and stromal cells [12]. In the 1990s, Helen D et al. reported that the prostatic OT increased epithelial cell growth and both muscular tone and contractile activity [13]. Later evidence also showed that OT, OT-associated neurophysin and the OT receptor (OTR) were expressed in prostatic stromal and epithelial cells [12]. During the prostatic disease process, increased prostatic OT expression was reported in BPH as well as prostatic intraepithelial neoplasia (PIN). In the invasive prostate cancer tissue, OT concentration in prostate seemed to be reduced [14, 15]. It is also thought that the OT proliferative effect on the prostate tissue may be mediated through the caveolae-associated receptors, a process which seems to be androgen receptor-independent [12].

Noteworthy, the oxytocin receptor (OTR) is a G protein–coupled receptor (GPCR), which activates the GTP-binding protein Gαq to stimulate phospholipase C_β_. Additionally, OTR can also couple to G_i_ and G_h_. GPCRs affect the progression of PCa in various ways according to the different types of GPCR [16]. Previous studies, have proved that OT stimulates PCa growth and that a Gi-dependent mechanism is involved in the OTR-mediated migration of prostate cancer cells. Some also argued that OT influences 3-hydroxysteroid dehydrogenase and 5-reductase activities in PCa cell lines and this effect may represent an important step of oxytocinergic regulation of steroidogenesis in PCa [17]. Besides, many GPCR tend to interact with APPL1, a protein that was originally treated as an Akt2-binding protein, whose unique structures include an adaptor protein with a pleckstrin homology (PH) domain, a phosphotyrosine binding (PTB) domain and a leucine zipper motif. APPL1 has the ability to interact with the tumor suppressor protein, deleted in colorectal cancer (DCC). In addition, 14 proteins can interact with it in various signaling pathways involved in cell apoptosis, proliferation and cell survival [18, 19]. Thus, APPL1 is a hotspot in the study of cancer.

In summary, our study confirmed the proliferative effect of OT on PCa *in vitro* and *in vivo* through the elevated expression of APPL1. PC-3 (the androgen-independent cell line) and LNCaP (the androgen dependent cell line) were cultured, both of which have been widely utilized in the study of PCa and both of them are adherent epithelial cells growing in aggregates [20]. Furthermore, we investigated, for the first time, the relationship between the levels of OT in serum and prostatic tissue and the development of PCa. Accordingly, we conclude that OT may be a novel target for PCa treatment.

## RESULTS

### Serum oxytocin level is elevated in the PCa patients

**Figure 1:**
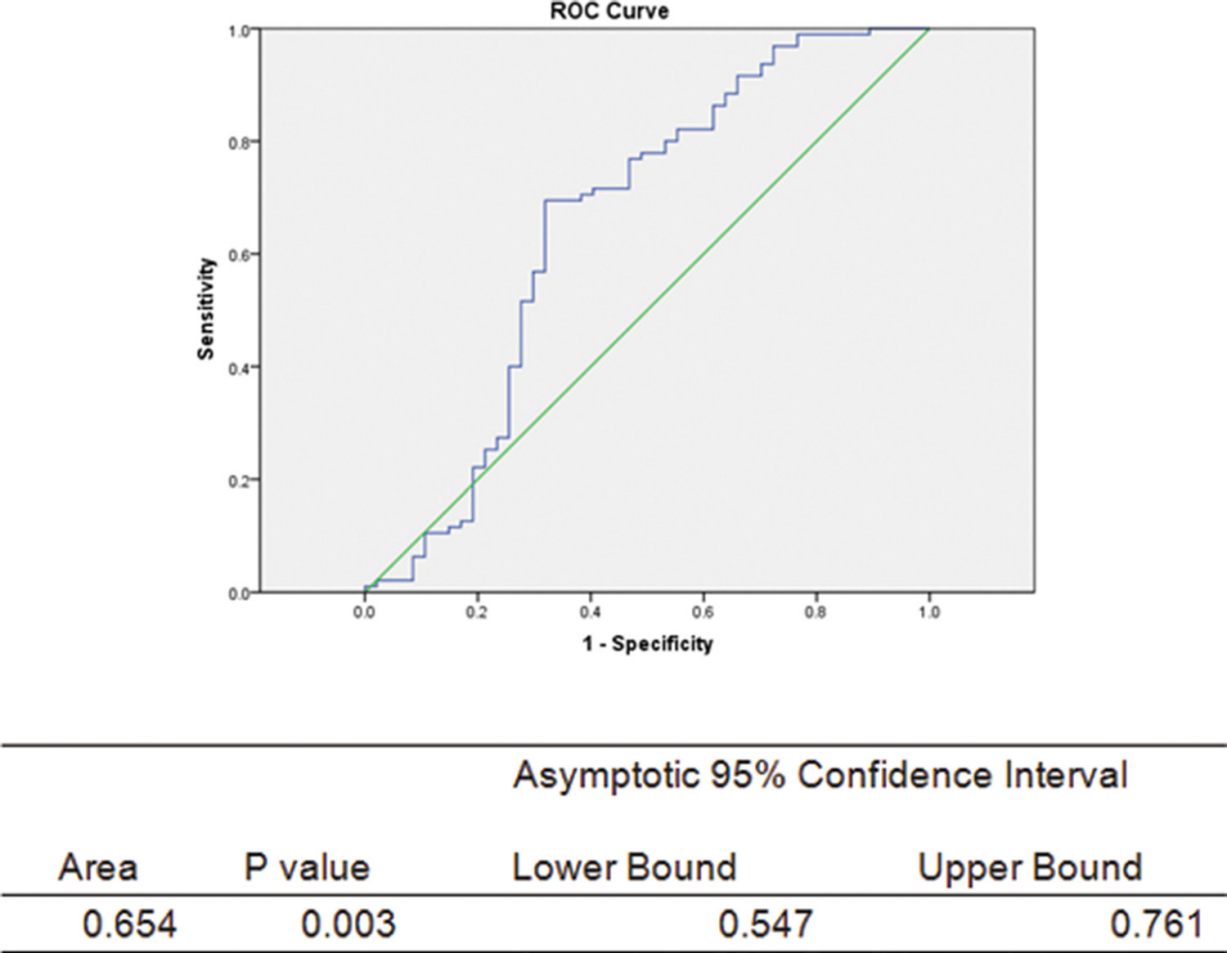
ROC curve for the prediction of PCa by serum oxytocin level The green middle straight line is the reference line. Abbreviations: CI = confidence interval, Area under the curve = AUC.

The basic patients’ serum parameters are shown in Table 1. In total, 51 normal or BPH patients and 98 PCa patients were included in the serum study. In the prostate tissue analysis, tissue samples from 206 patients were analyzed, including 101 prostate cancer patients, 98 BPH patients and 7 healthy individuals included in the tissue microarray or obtained from our tissue bank as described above. Patients were grouped into the PCa group and the non-PCa group. The results revealed that the OT serum level was significantly up-regulated in the PCa patients (31.31 ± 13.34 vs. 37.53 ± 9.18, *P* = 0.005). In order to exclude the confounding factors, such as the metabolic element, logistics analysis was applied. The results of this analysis are shown in Table 3. OT was not significantly elevated in PCa patients, with the analysis of PSA (*P* = 0.075) and CHO (*P* = 0.093) being adjusted. Furthermore, the receiver operating characteristic (ROC) curve was also plotted, as shown in Figure 1.

**Table 1:** Basic characteristics of involved individuals in the serum study

	Health patients /BPH (*N* = 51)	PCa (*N* = 98)	*P* value
Age	67.63 ± 5.03	67.83 ± 4.82	0.81
PSA	2.18 ± 2.42	35.94 ± 99.31	0.001**
fPSA /PSA	0.40 ± 0.49	0.07 ± 0.07	< 0.001**
BMI	22.07 ± 1.18	22.05 ± 1.11	0.94
BG	5.43 ± 0.32	5.27 ± 0.47	0.01*
SP	125.63 ± 13.26	127.51 ± 12.78	0.40
DP	79.02 ± 10.19	77.95 ± 10.11	0.54
CHO	3.98 ± 0.77	4.71 ± 0.92	0.01*
TG	1.44 ± 0.79	1.57 ± 0.73	0.63
LDL	2.59 ± 0.63	3.18 ± 0.63	0.01*
HDL	1.16 ± 0.31	1.12 ± 0.43	0.73
FFA	0.38 ± 0.15	0.35 ± 0.13	0.46
OT	31.31 ± 13.34	37.53 ± 9.18	0.005**

**Table 2:** The clinical and pathological features of PCa patients and normal individuals (n = 206)

	Normal (*N* = 105)	PCa (*N* = 101)
Age	68.00 ± 9.75	69.20 ± 7.39
Sex	Males	Males
Gleason score	-	6 (5–6)
TNM	-	I (15.8%)
	-	II (41.6%)
	-	III (27.7%)
	-	IV (14.9%)
OT expression	70.04 ± 14.61	95.68 ± 32.93*

The data of age and OT expression are shown as means ± SD. The gleason score is shown as the median (quartiles).

**Table 3:** Univariate and multivariate logistics regression for risk of PCa in relation to serum oxytocin levels

Model	OR (95% CI)	*P* value
OT univariate	1.06 (1.02–1.10)	0.002
OT, BG adjusted	1.05 (1.02–1.09)	0.002
OT, PSA adjusted	1.07 (0.99–1.14)	0.075
OT, LDL adjusted	5.10 (1.22–21.33)	0.026
OT, CHO adjusted	1.05 (0.99–1.12)	0.093
OT, multiple adjusted#	1.15 (0.93–1.43)	0.194

#Multivariate model included: BG, PSA, LDL, CHO.

Abbreviations: BG = blood glucose, PSA = prostate specific antibody, CHO = cholesterol, LDL = low density lipoprotein, OT = oxytocin.

### Oxytocin and its receptor are highly expressed in the PCa tissue

**Figure 2:**
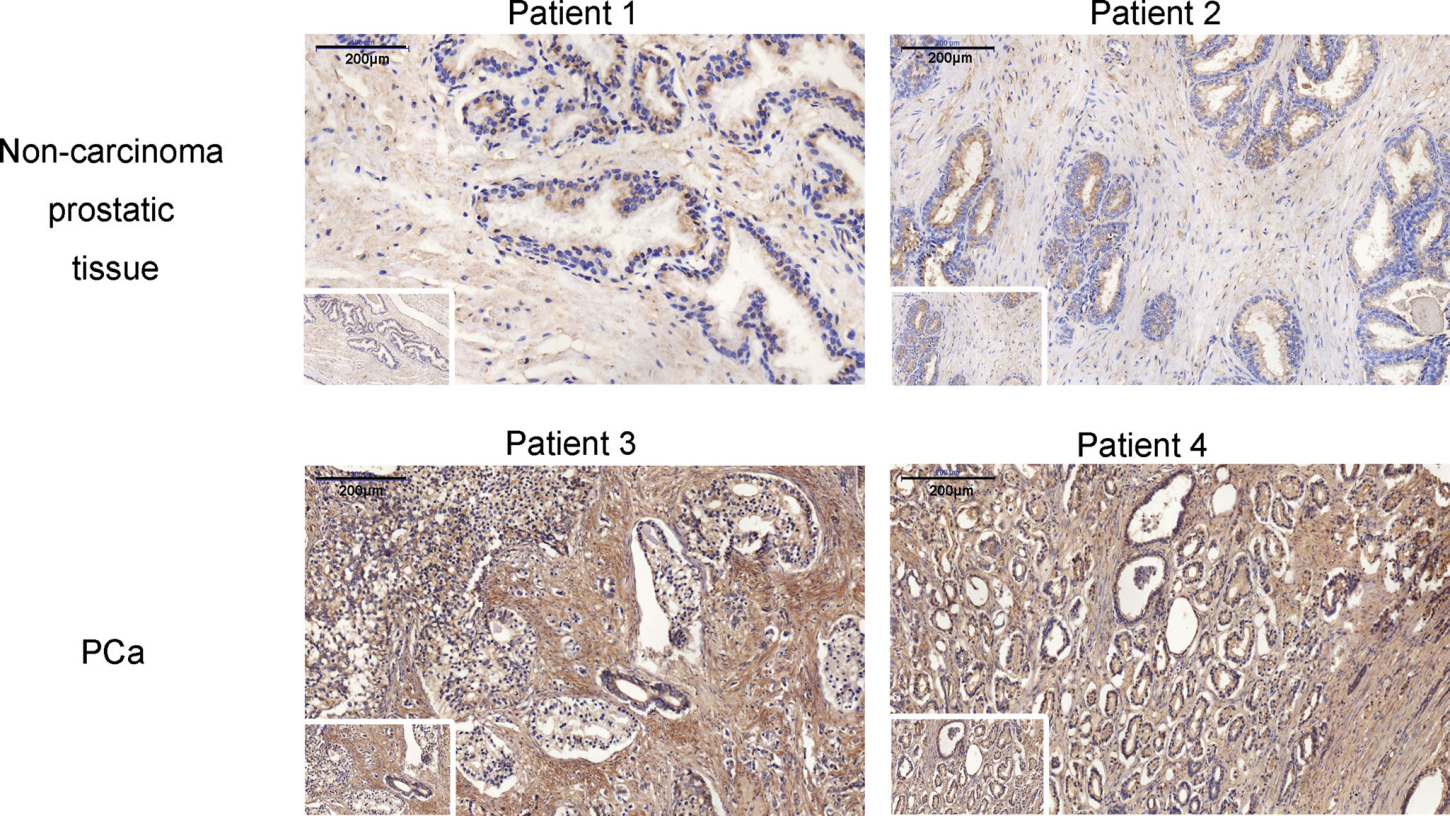
The oxytocin expression in no-carcinoma prostate tissue and the PCa tissue The expression is significantly elevated in the PCa tissue. Views in the white frame are magnified 100 times and views in the big frames are magnified 200 times. Scale bar = 200 μm.

**Figure 3:**
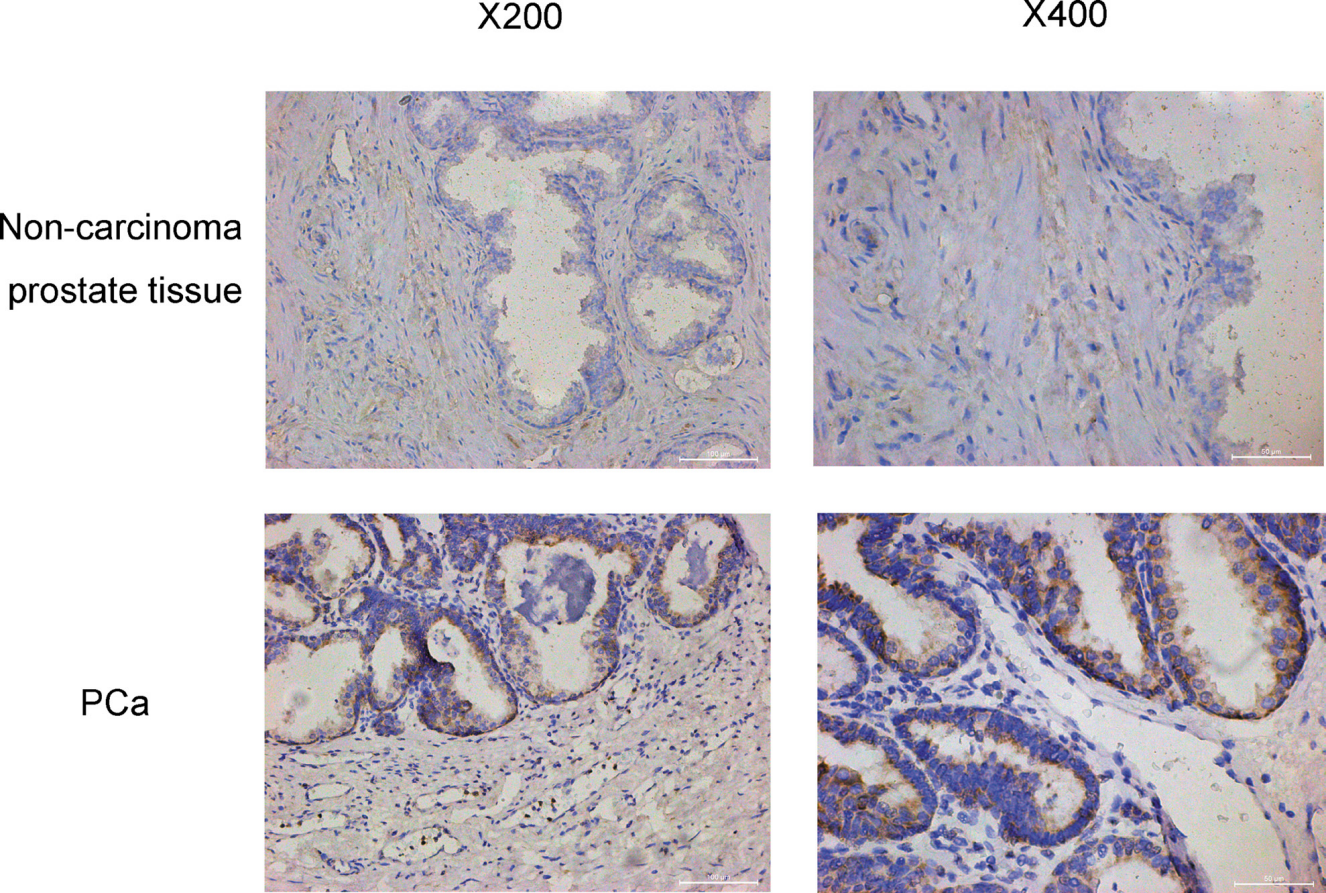
The oxytocin receptor expression in no-carcinoma prostate tissue and the PCa tissue The expression is increased significantly in the PCa tissue. The views on the left are magnified 200 times and the views on the right are magnified 400 times.

The clinical and pathological features of PCa patients and non-carcinoma individuals are presented in Table 2. The immunohistochemical analysis showed an elevated OT and OTR expression levels in the PCa tissue compared with the tissues of non-carcinoma subjects (Figures 2 and 3). The OT expression analysis revealed that OT expression was mainly elevated in the prostate cells, as shown in Figure 2 (83.00 (78.25–90.42) vs. 93.29 (72.26–112.98); *p* = 0.026). However, no significant difference was observed among the different Gleason grades of the PCa progression (Spearman analysis, correlation coefficient = –0.035, *N* = 93, *p* = 0.741). In the TNM classification analysis, OT was shown to be elevated with the progression of the carcinoma (Spearman analysis, correlation coefficient = –0.216, *N* = 93, *p* = 0.037).

### Oxytocin increases proliferation and decreases apoptosis of PCa cells

**Figure 4:**
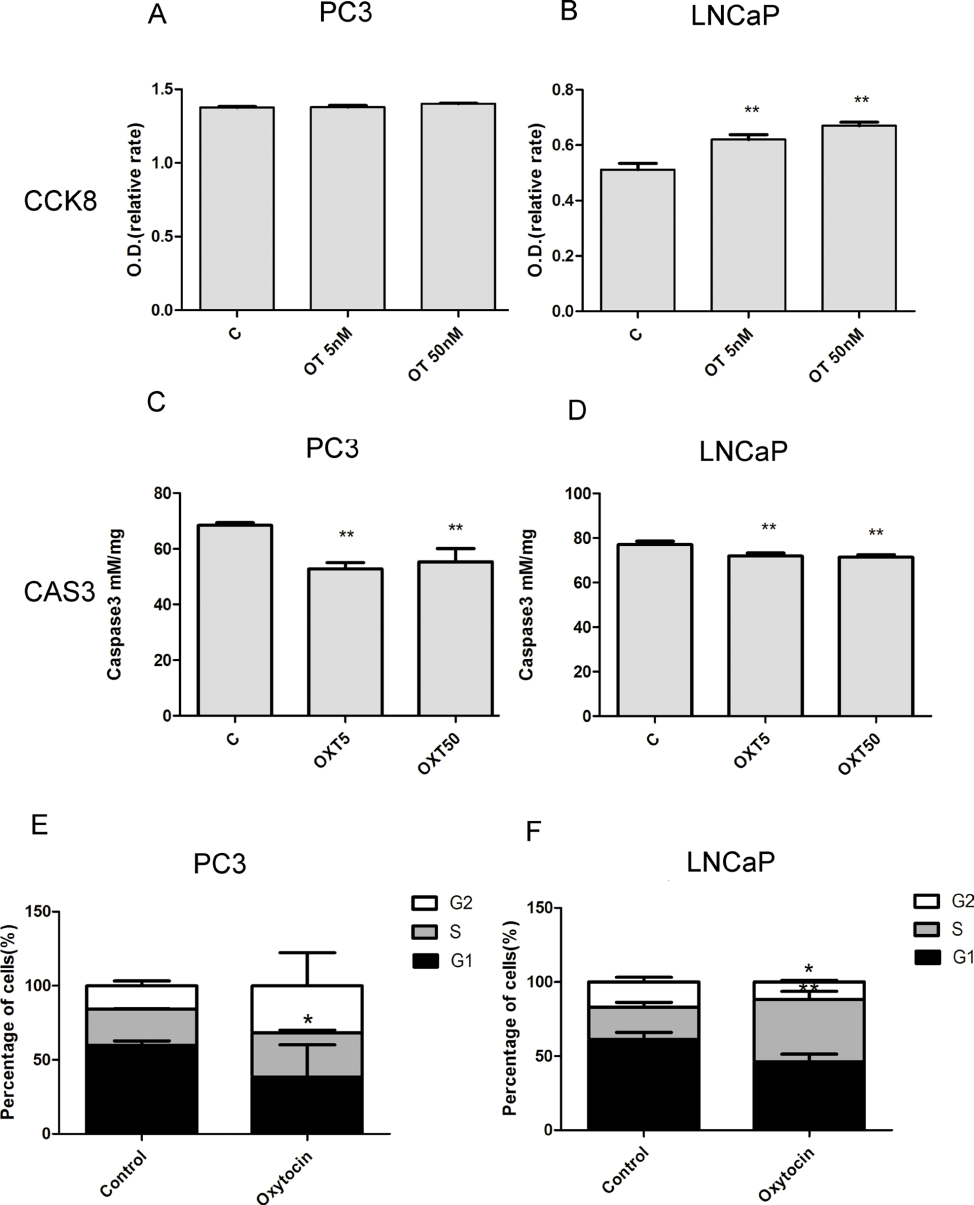
Oxytocin (5 nmol/L; 50 nmol/L) increases the proliferation of LNCaP cells and reduces the apoptosis of the PC3 and LNCaP cells (**A, B**) Oxytocin elevates the proliferation of LNCaP and no significant difference is observed in the PC3 cell line. (**C, D**) Oxytocin reduces apoptosis of the LNCaP and PC3 cell lines in both doses. (**E**) In the PC-3 cell line, oxytocin increase the average proportion of S phase from 24.68 ± 0.26% to 29.85 ± 1.88% (*p* < 0.05). (**F**) In the LNCaP cell line, oxytocin tends to decrease the average proportion of G0/G1 phaseand and increase the average proportion of S phase from 21.72 ± 3.45% to 42.09 ± 5.43% (*p* < 0.01) significantly. All The values are shown as the means ± SD. *P <0.05, **P < 0.01 (compared with the control group); *N* = 3-5. Abbreviations: C: control, OT: oxytocin, CAS3: caspase3.

**Figure 5:**
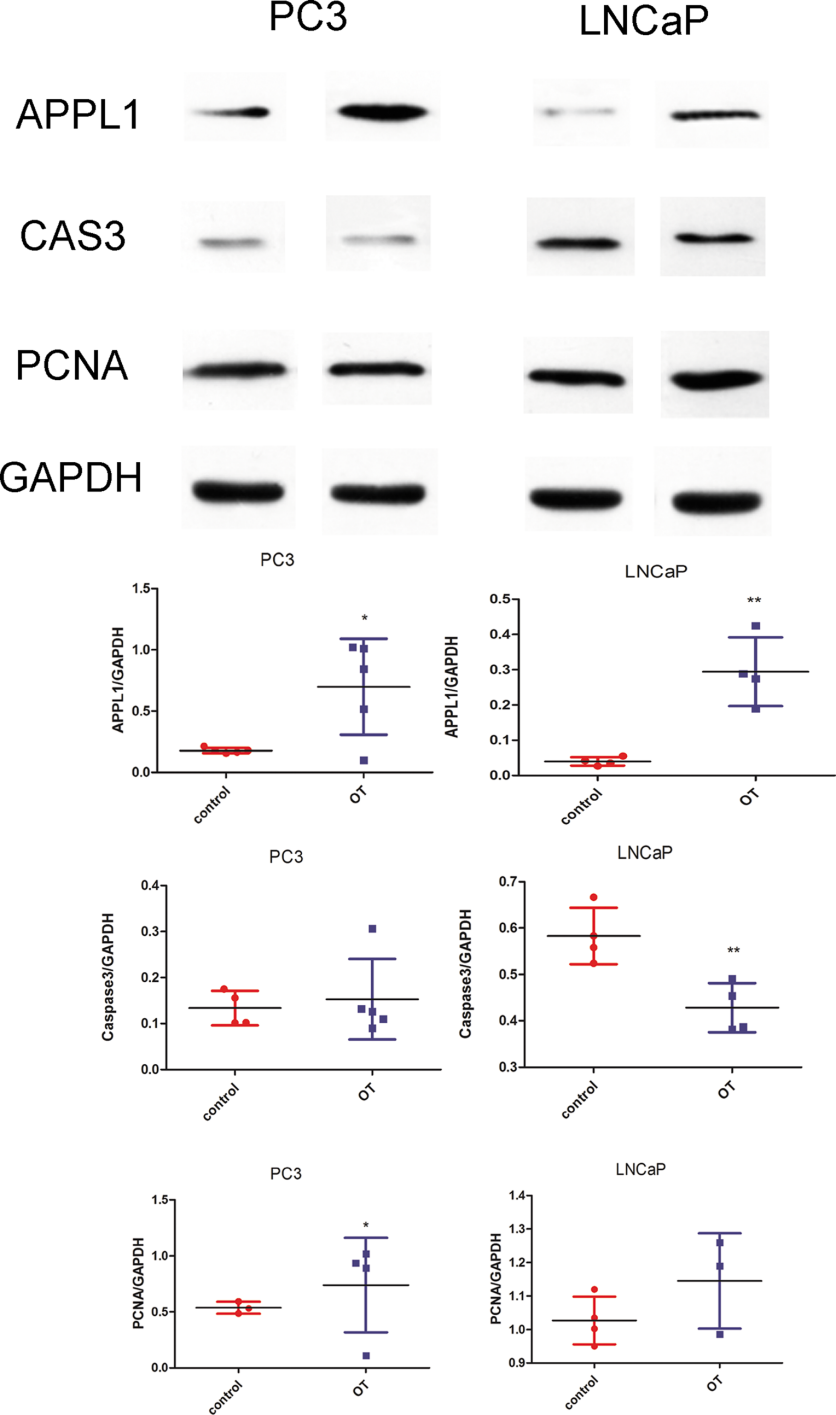
Western blot analysis of the cell markers in the PC3 and LNCaP cell lines Oxytocin elevates APPL1 expression in PC3 and LNCaP cell lines. Caspase-3 was significantly decreased in the LNCaP cells by oxytocin. In the PCNA analysis, oxytocin increases the expression in PC3 cells significantly. All the values are shown as means ± SD. *P < 0.05, **P < 0.01 (compared with the control group) OT: oxytocin (50 nM) treated group; C: control group which was treated with PBS.

In the cellular study, analysis of cell proliferation with the CCK8 assay showed that oxytocin increased the proliferation of LNCap cells, but no significant effect was observed in the PC3 cell line (Figure 4). In the analysis of the caspase3 activity, it was found that caspase3 activity was significantly reduced in both the PC3 and LNCap cells. Western blot analysis also confirmed these results, as shown in Figure 5. After the treatment with oxytocin, caspase3 expression was significantly reduced, whereas PCNA expression was highly elevated. In addition, APPL1 was also significantly elevated after the OT treatment. The proliferative effect was also confirmed by the cell cycle analysis. In the PC-3 cell line, with the treatment of oxytocin, the average proportion of G0/G1 phase tended to be decreased from 59.56 ± 3.28% to 38.29 ± 21.81% (*p* > 0.05) and the average proportion of S phase increased from 24.68 ± 0.26% to 29.85 ± 1.88% (*p* < 0.05). In the LNCaP cell line, oxytocin tended to decrease the average proportion of G0/G1 phase from 61.16 ± 4.81% to 46.15 ± 5.17% (*p* > 0.05) and the average proportion of S phase increased from 21.72 ± 3.45% to 42.09 ± 5.43% (*p* < 0.01) significantly.

## DISCUSSION

In this study, serum OT level was shown to be elevated in PCa patients compared with the levels in no-carcinoma patients. In the tissue immunohistochemistry analysis, the expression of OT and its receptor were both elevated in the PCa tissue samples, though no significant difference was observed among PCa tissue samples of various gleason grades, which is the first time this is reported. Furthermore, the proliferative effect was observed *in vitro* in the cell culture experiments and APPL1 was also found to be highly expressed after the treatment with OT.

Expression of OT has been extensively investigated in prostate tissues and cell lines [12, 21]. OT mRNA was found to be present in all prostate cell lines examined, including PrSC, PrEC, RWPE1, RWPE2, LNCaP, DU145, PC3 and PC3M [16]. Higher level of OT was detected in the prostate hyperplasia and prostatic intraepithelial neoplasia (PIN) [12]. However, as was reported, oxytocin expression in carcinoma was reduced with tumour progression, which is studied by Whittington K et al. in invasive protate cancer tissue [12]. The differences may be caused by the numbers involved in study. Moreover, the difference may be also caused by the neoadjuvant therapy, as for most of our included surgical patients neoadjuvant therapy was used during our clinical treatment. As was reported, prostatic oxytocin concentrations can be decreased by testosterone and increased following castration or treatment with an anti-androgen [22]. In the paper of Whittington K, they reported the intensity of staining in areas of PIN varied between samples and, in some cases, was stronger than that seen in tissue exhibiting BPH [12]. Among our results, interestingly, there is no significant correlation between oxytocin expression and gleason scores (*p* = 0.741). In the TNM classification, however, significant negative correlation exists between them (*p* = 0.037).

Furthermore, our previous report proved the proliferative role of OT in prostate enlargement both in the animal model and the *in vitro* study. Regarding prostate diseases, increased expression of the OTR was also found in prostate neoplastic epithelial cells compared to hyperplastic cells, though it was highly expressed in the hyperplasic prostate tissue compared with the normal tissue [12]. This result was consistent with the OT level and may suggest a potential autocrine or maybe paracrine role of OT in functions of the human prostate. Many studies have reported higher levels of OTR in all prostate cancer cell lines though not significant difference was observed between these prostatic cancer cells [16]. This study confirms these results through the tissue immunohistochemical analysis and for the first time proved the elevated OT level in the PCa serum. Therefore, both OT and OTR show increased expression in prostate carcinomas, which indicates its important role in prostate diseases. However, in our study, no significance was observed between gleason scores and oxytocin expression.

In the prostate tissue, OT has been shown to participate in the proliferative process both in epithelia cells as well as stromal cells. It has also been reported that in the prostate, the oxytocinergic system increases the smooth muscle contractile activity [23] and prostate contraction during ejaculation [24]. Furthermore, OT is involved in regulating the conversion from testosterone to dihydrotestosterone by differential regulation of 5AR I/II [25]. It is also known that oxytocin stimulates steroidogenesis in several organs by modulating activity of 3-hydroxysteroid dehydrogenases and steroid 5-reductases in both the androgen-dependent LNCaP and androgen-independent PC-3 human prostate cancer cell lines [26]. Thus, OT contributes considerably to the prostatic disease development. As a hypothalamus hormone, one of the important roles of OT is energy regulation and improvement, especially in the lipid metabolism. Apart from the central regulation of food intake and neuron interaction, it regulates lipid metabolism through peripheral ways involved in liver, fat tissue and muscle [27, 28]. Besides, metabolic disorder has been widely accepted to be involved in the pathogenesis of PCa. Epidemiology and animal studies have detected the proliferative effect of metabolic disorders on carcinoma [29]. Accordingly, OT may also be one of the key factors involved in the regulation of metabolic disorder-induced PCa.

Carcinoma research has revealed that OT functions to regulate tumor progression in several types of cancers, such as rectal cancer, breast cancer, lung cancer, etc. [30–32]. OT tends to act as a mitogenic factor for the growth of Kaposi's sarcoma, but does not participate in tumor migration or angiogenesis. On the other hand, OT also induces cell proliferation and migration of human endothelial cells derived from breast carcinoma [32, 33]. Hence, OT appears to be involved in many biological characteristics. OT is reported as a growth factor, whether activator or inhibitor. Interestingly, it is believed that OT is a protective hormone against breast cancer and OT can inhibit the progression of ovarian carcinoma cells both *in vitro* and *in vivo* [34].In a study on PCa, OT tended to improve the growth and migration of PCa cells [16]. In this study, we mainly focused on the proliferation and growth effect of OT on PCa. A proliferative effect was observed in the cell culture study, which was consistent with the results of the serum analysis. As an important regulator for cancer development, G protein-coupled receptor (GPCR) plays important roles depending on the various protein types interacting with it. In certain carcinoma, some kinds of GPCRs and G proteins may play tumor suppressive roles, while its mutation may reflect inactivation of these genes. For instance, in the androgen receptor signaling pathways, the function of GPCR differs due to the diverse subunits. It affects chemotaxis through the MEK-ERK pathway, survival through the PI3K-Akt pathway and causes cell death through the p38 pathway [35]. Thus, the performance of GPCR involved in BPH or PCa are different depending on the diverse cellular signaling involved. OTR couples to multiple G-proteins including G_q_ and G_i_. In turn, these G-proteins may convey the OT signaling to different intracellular pathways leading to distinct cellular functions. Chemotaxis has been associated with activation of GPCRs that couple exclusively to Gi. As is known, OT would induce migration of prostate cancer cells if endogenous OTRs couple effectively to the G_i_-dependent pathway [36].

Interestingly, APPL1, as an adaptor protein with an important role in tumor promotion, has been reported to mediate adiponectin signaling by binding to adiponectin receptors. Previous studies observed high APPL1 expression in C2C12 myotubes, insulinoma cells, L6 cells; moderate expression was detected in HEK293, mouse hepatocytes, while low expression was found in mouse brain, skeletal muscle, fat, heart, spleen and to a lesser extent, pancreas and kidney tissues [37]. Expression of APPL1 is correlated with clinicopathologic characteristics and poor prognosis in patients with gastric cancer [38]. In PCa study, APPL1 has also been shown to play an important role. In addition, APPL1 has been shown to be involved in the cell metabolism, including lipid oxidation and glucose uptake, through the insulin signaling pathway and the translocation of the glucose transporter, which activate the AMP-activated kinase, p38, MAPK, and PPAR. Moreover, it reduces the endosome relocalization through the APPL1-PI3K-Akt pathway [39]. Its increased expression in the PCa contributes to the uptake of glucose and induces tumor growth [40]. The APPL1-PI3K-Akt pathway has also been reported to mediate the decrease of androgen receptor transactivation. As a negative regulator, APPL2 competes for binding with APPL1 to regulate energy metabolism and the other pathways mentioned above. In our study, APPL1 was found to be increased and mediated the proliferative effect of OT on PCa, and maybe through an APPL1 regulation pathway.

Furthermore, as has been suggested, numerous endocrine peptides contribute to the development and migration of PCa, such as androgen, parathyroid hormone, etc. Indeed, the prostate can be considered as a sexual gland which is greatly affected by endocrine hormones [41]. In addition, the hypothalamus is the most important organ regulating the endocrine system through both central and peripheral pathways; the endocrine system itself plays an important part in the morbidity of PCa. Widely used in clinical work, gonadotropin-realizing hormone (GnRH) analogues, including the luteinizing hormone-releasing hormone (LHRH) agonists and GnRH antagonists, are the most common agents used for medical androgen deprivation in the treatment of PCa [42]. Moreover, consistent with our present work, OT acts as a potent growth factor in PCa. In this way, through these hormones, the hypothalamus may participate in the pathological process of PCa. It may be named as the pituitary–testis–prostate axis or hypothalamic–pituitary–gonadal axis. In addition, regarding the central regulation, some studies have been published indicating that behavioral stress accelerates PCa development in mice. This function is mediated through the activation of the adrenaline/ADRB2/PKA/BAD anti-apoptotic signaling pathway. Therefore, sympathetic nervous system (SNS), whose central regulator is the hypothalamus, which tends to be involved in PCa progression [43]. To sum up, the hypothalamus-prostate regulation tends to be an important regulation system of prostate diseases, such as PCa. Additional medical research may be launched based on this regulation system in future studies.

Our study also has several limitations. First, very small numbers of patients were involved in this retrospective study and large scale study should be launched to improve the reliability of the results. In the serum analysis study, the TNM and the Gleason scores did not be well analyzed according to the limited amount of individuals in this study. Second, more signaling details should be analyzed in the future studies. Third, in future studies gene knockdown cells and animal studies will be necessary to validate these results. Fourthly, as the follow-up data of patients were not available, survival curve cannot be summarized. Fifthly, further studies may be done to test the details of apoptosis process as no distinction of the early stage or late stage was detected in this study.

In conclusion, serum OT may be a biomarker for PCa diagnosis and progression. Additionally, expression of prostatic oxytocin and its receptor were significantly increased in PCa patients. Moreover, OT increases prostate cancer growth and induces the expression of APPL1. Thus, oxytocin may be involved in the the hypothalamus-prostate regulation system of PCa development.

## MATERIALS AND METHODS

### Study population

This was a clinical retrospective cohort study performed at our department using a serum and tissue bank collected from August 2015 to July 2016. The patients were systematically diagnosed with PCa based on the pathological analysis of their tumor. The exclusion criteria were as follows: patients with severe cardiovascular, pulmonary or renal disease, bleeding disorders, type 2 or type 1 diabetes, hyperlipidemia, severe obesity (BMI ≥ 30 kg/m^2^), poor blood pressure control, bladder cancer, previous prostate surgery and the use of PCa neoadjuvant therapy. Ethical approval and the written informed consent of the patients were obtained. Blood samples were collected after overnight fasting in EDTA-containing tubes and centrifuged to obtain the serum samples, which were frozen at –80°C until use. Other clinic characteristics, including the past medical history, blood pressure, MRI analysis, body height, weight, etc. were recorded at the patients’ hospital visit.

The patients’ blood samples were collected and separated by centrifugation (2500×g, 5 min). The parameters, including prostate specific antigen (PSA), glucose, triglycerides, total cholesterol, high-density lipoprotein (HDL)-cholesterol and low-density lipoprotein (LDL)-cholesterol, were analyzed using commercially available spectrophotometric assay kits (Sigma-Aldrich, Saint Louis, MO, USA). Serum OT level was measured using the OT ELISA kit (Shanghai Enzyme-linked Biotechnology Co., Ltd., Shanghai, China) following the manufacturer's instruction.

### Oxytocin expression in prostate cancer

Expression of OT and OTR was determined in prostate tissue from our tissue bank and the tissue microarray (PR803c) was obtained from US Biomax Inc. (Rockville, MD, USA), which together comprised 7 normal prostatic tissue cores, 98 BPH tissue samples (all were treated as no-carcinoma tissue) and 101 prostatic cancer tissue cores. IHC staining was performed using antibodies against OT (ab2078, 1:200; Abcam, Shanghai, China) and OTR (ab115664, 20–40 μg/ml; Abcam). First, hematoxylin and eosin (HE) staining was used to visualize the gross tissue morphology. The secondary antibody was a goat anti-rabbit IgG (sc-2040; Santa Cruz Biotechnology Inc., Santa Cruz, CA, USA) used at a 1:200 dilution. Hematoxylin was further counterstained for the nuclei. All the areas with the most intensity were evaluated by scanning at two different magnifications. H-Score of each core was calculated with the QuantCenter software (version 2.0, 3DHISTECH Ltd., Budapest, Hungary) which can recognize and analyze the staining intensity (0, negative; 1 weak; 2 moderate; 3 strong) and positive staining area (pixels). The H-Score was calculated as follows: H-Score=∑ (PI×I) = (percentage of cells of weak intensity ×1) + (percentage of cells of moderate intensity ×2) + (percentage of cells of strong intensity ×3).

### Cell culture

Two kinds of prostatic cancer cell lines were used in our study, namely the PC3 and the LNCaP human prostate cancer cell lines (ATCC, Manassas, VA, USA) and maintained in prostate epithelial cells medium (PEpiCM; ScienCell Research Labs., Carlsbad, CA, USA) with 1% prostate epithelial cell growth supplement (PEpiCGS; ScienCell Research Labs.) and 1% penicillin/streptomycin solution (P/S, ScienCell Research Labs.) or grown in high-glucose DMEM (HyClone Labs., Logan, UT, USA) supplemented with 10 % fetal bovine serum (FBS, HyClone Labs.) and 1 % penicillin/streptomycin. During the experiment, the cell were treated with two different concentrations of OT(5 nmol/L and 50 nmol/L) for 90 min.

### CCK-8 proliferation and Caspase-3 activity assay

Cell proliferation was assessed by the CCK-8 assay (Beyotime, Shanghai, China, C0037) following to the manufacture's instruction. Cells were seeded into 96-well plates and cultured for an additional 24 h. After treatment with OT, 10 μl of the kit reagent was added and then incubated for another 2 h. O.D. value was read at 450 nm to obtain the final results.

Cell apoptosis was partly detected by a caspase-3 assay (ab39401; Abcam) using a colorimetric method following the manufacture's instruction. Samples were incubated at 37°C for 90 min and optical density (O.D. = 400 nm) was measured to determine the levels of caspase-3.

### Cell cycle analysis

Plated in the culture dishes at density of 1 × 106 cells per dish, cells were starved for 12 h and for treated conditions for another 12 h. The single cell suspension was fixed with 70 % cold ethanol for 12 h. Cells were then stained with propidium iodide (PI) mixture for 30 minutes at 37°C before flow cytometry analysis (Beckman Coulter Flow Cytometer, Krefeld, Germany) following the manufacturer's instructions (C1052, Beyotime Biotechnology, China). Cell cycle distribution was further analyzed with ModFit LT (V4 1.7, Verity Software House, Topsham, ME).

### Western blot analysis

Treated cells were lysed in cold RIPA lysis buffer and protease inhibitor cocktail followed by centrifugation at 12,000 × g for 20 min at 4°C. We used the primary antibodies against the OTR (ab181077, 1:1000; Abcam), PCNA (ab18197, 1 μg/m; Abcam), caspase3 (ab13847, 1:500; Abcam), cleaved caspase-3 (9661, 1:1000; CST), APPL1 (ab59592, 1:500; Abcam), GAPDH (5174, 1:1000; CST). The secondary antibody was peroxidase-conjugated IgG (Beijing Zhongshan Jinqiao Biotec) used at a 1:8000 dilution. The intensity of the bands was analyzed using the Quantity One software (Bio-Rad, Hercules, CA, USA).

### Statistics

Patient characteristics are reported as means ± standard deviation (SD) and analyzed using the *t-test*. Gleason scores were showed as medians and interquartile range (IQR). To study the confounding factors, multivariate logistic regression models were calculated by stepwise selection of the most significant predictors on univariate analysis. For the analysis of the immunohistochemistry, Wilcoxon test was ustilized to test the significance. All data from cell culture are presented as the means ± SD; statistically significant differences were assessed by analysis of variance (one-way ANOVA). A two-sided *P* < 0.05 was considered statistically significant. All the above data were analyzed using the SPSS software version 19.0 (IBM Corp., Armonk, NY, USA).
